# Determination of factors affecting medication adherence in type 2 diabetes mellitus patients using a nationwide claim-based database in Japan

**DOI:** 10.1371/journal.pone.0223431

**Published:** 2019-10-08

**Authors:** Takeshi Horii, Kenji Momo, Takeo Yasu, Yusuke Kabeya, Koichiro Atsuda

**Affiliations:** 1 Research and Education Center for Clinical Pharmacy, Division of Clinical Pharmacy, Laboratory of Pharmacy Practice and Science 1, Kitasato University School of Pharmacy, Kanagawa, Japan; 2 Department of Pharmacy, The Research Hospital, The Institute of Medical Science, The University of Tokyo, Tokyo, Japan; 3 Department of Hospital Pharmaceutics, School of pharmacy, Showa University, Tokyo, Japan; 4 Department of Medicinal Therapy Research, Pharmaceutical Education and Research Center, Meiji Pharmaceutical University, Tokyo, Japan; 5 Department of Home Care Medicine, Sowa Hospital, Kanagawa, Japan; Fordham University, UNITED STATES

## Abstract

**Background:**

The extent of medication adherence in patients with type 2 diabetes mellitus (T2DM) several years after starting treatment with hypoglycemic agents remains unknown. Most previous work on medication adherence targeting this group of patients has been undertaken across a single year or is questionnaire based. This study aimed to determine medication adherence status and factors affecting adherence 3 years after initiation of hypoglycemic agents, using a nationwide medical claim-based database in Japan.

**Methods:**

This retrospective study was conducted on data from 884 subjects with T2DM to better understand medication adherence, the effects of polypharmacy, and other factors. We also investigated the effects of medication nonadherence on hemoglobin A1c levels. Proportion of days covered was defined as the number of days for which a hypoglycemic agent was prescribed and in the patient’s possession to the number of days in the observation period. A proportion of days covered ≥0.8 were considered adherent, and those with a value <0.8 as nonadherence. Polypharmacy was defined as taking ≥5 medications.

**Results:**

Of the 884 patients investigated, 440 were considered adherent during the study period. Significant factors related to adherence included number of medications (3 or 4, or ≥5), male sex, age 50–<60 years, and total number of visits ≥17. Medication adherence was also a factor related to patients with hemoglobin A1c values < 7.0% at the end of the observation period.

**Conclusions:**

We surveyed medication adherence for 3 years with post medication initiation, and found that subjects aged 50–<60 years, those with ≥3 concomitant medications, and those with a total number of visits ≥17 were more likely to be adherent and persistent, and more likely to continue their hypoglycemic agents. A high degree of medication adherence was found to have a positive influence on hemoglobin A1c levels.

## Introduction

Medication adherence refers to the extent to which patients take their medication regimen as prescribed by their health care provider [1]. A previous study suggests that about a quarter of patients are nonadherent, and that rates of adherence are higher among patients with acute conditions than in those with chronic conditions [2]. Even in the resource-intensive setting of clinical trials, the average adherence rates for trial drugs used in chronic diseases are between 43% and 78% [3–5]. A systematic review of patients with type 2 diabetes mellitus (T2DM) who remained on treatment with oral hypoglycemic agents for 6–12 months reported adherence rates of between 36% and 93% [6].

Medication adherence has been recognized as key for optimally controlled diabetes [7] in patients with T2DM. Several factors, such as disease and treatment characteristics and complexity, age, sex, stress, depression, and multidrug combinations affect treatment adherence amid changing circumstances in the daily lives of patients with diabetes [8]. When selecting an oral hypoglycemic agent, not only medical outcomes but also the patients’ quality of life and attitude toward treatment should be considered. Maintaining good glycemic control is believed to allow patients to maintain insulin production and reduce insulin resistance. Poor medication adherence makes achieving good glycemic control difficult, which is believed to affect the onset of diabetic microangiopathy (retinopathy, nephropathy, and neuropathy) and increase the risk of diabetic complications [9].

Most studies to date on medication adherence in patients with T2DM were conducted for a maximum of 1 year, with the impact of most of the interventions being assessed for only 6 months [10]. However, once treatment with a hypoglycemic drug is initiated, multi-year drug adherence investigations are needed, since most patients continue to take them for several years after diagnosis.

We conducted a 3-year retrospective study, using data on itemized medical claims, to determine the current status of medication adherence among patients with T2DM. The potential effects of polypharmacy and other factors on medication adherence in these patients were also assessed. We also investigated the effects of medication nonadherence on hemoglobin A1c (HbA1c) levels at the end of the 3-year observation.

## Methods

### Study design and data source

We conducted a retrospective study using an itemized medical claim database maintained by the Japan Medical Data Center. This database includes employment medical claims and medical check-up data provided by multiple health insurance societies [11,12]. This itemized medical claim database includes baseline patient attributes, the disease code (according to the Medical Information System Development Center Standard Disease Nomenclature Master and the International Classification of Diseases (ICD)-10), prescription details, and medical procedures.

### Subjects

The subjects in our study were patients who had visited our institute between May 2005 and January 2013, and fulfilled the following selection criteria: (i) a diagnosis of T2DM (ICD-10: E11) or unspecified diabetes (ICD-10: E14); (ii) a history of being prescribed a hypoglycemic agent; and (iii) data available for 3 years of continuous follow-up. Exclusion criteria included: (i) disease name/code for type 1 diabetes; and (ii) age either <18 years or ≥75 years. The index date was defined as the month during which the prescription for the hypoglycemic agent was issued.

### Definition of medication adherence

We utilized the proportion of days covered (PDC) as our index of medication adherence, which we calculated for each patient. PDC is defined as the number of days for which the hypoglycemic agent was prescribed and in the possession of the patient, to the number of days in the observation period:

PDC = [no. of days for which the hypoglycemic agent was possessed during the observation period (days)/observation period (days)]

Patients were categorized in the good adherence group if they achieved a PDC threshold of at least 80% [13–16]. A PDC value of ≥0.8 was considered adherence, and of <0.8 was considered nonadherence.

### Definition of polypharmacy

As described in a previous study on polypharmacy [17], we defined polypharmacy as the use ≥5 drugs, after excluding the following medications: injectable drugs, external use drugs, antibiotics, and any other drugs used in the treatment of non-chronic diseases, such as infections, prescribed for a total continuous use of less than 7 days. The number of drugs that were prescribed at the start of the observation period was used to determine polypharmacy.

### Patient characteristics

Age, sex, and body mass index (BMI) were included as baseline characteristics and were identified using the data listed on the claims in the same year as the index date occurred. Data on HbA1c levels were collected from claims listed in the same year as the month of initiation of the observation period, and claims listed in the same year were those received in each of the 3 years observed after that. The total number of visits to our institute for the purpose of diabetes treatment during the 3-year observation period (the total number of visits) was calculated by counting the number of days on which the subject was prescribed the hypoglycemic agent during that period. Target values for blood glucose control and weight control in diabetic patients was set at HbA1c <7.0% for the former and BMI <25 kg/m^2^ for the latter as recommended by the Japan Diabetes Society guidelines.

### Statistical analysis

Patient data were expressed as mean ± standard deviation (SD). Continuous variables were analyzed using unpaired t-test. Categorical variables were analyzed using chi-square test and are expressed as absolute numbers or percentages. Factors related to adherence at the end of the observation period were examined using logistic regression analysis. The number of oral medications (1, 2, 3, 4, ≥5), sex, age at baseline (<40, 40–49, 50–59 and ≥60 years), HbA1c levels (cut-off value 7.0%), BMI (cut-off value 25 kg/m^2^), use of hypoglycemic agents, and the total number of visits with cut-off values calculated using receiver operating characteristic (ROC) analysis were used as explanatory variables.

At the end of the observation period, factors related to maintaining HbA1c values <7.0% were analysed. These factors included the abovementioned explanatory variables and medication adherence. Univariate analysis was conducted for each explanatory variable and factors for which *P* <0.2 were included in a multivariate analysis to calculate the odds ratio (OR). Differences were regarded as significant when *P* <0.05. All statistical analyses were performed using the Stata software program (version 10; StataCorp, College Station, TX, USA).

### Ethical considerations

Because the data were retrospective, deidentified, and anonymous, the institutional review board committee of Ktasato University determined that this study did not constitute research with human subjects. It was therefore exempted from institutional review board consideration.

## Results

There was a total of 884 subjects, and their background characteristics are shown in Table 1. We compared the patients’ background characteristics after dividing the subjects into adherent and nonadherent groups. Significant differences (adherent vs. nonadherent) were found in the following: number of medications (2.6 ± 2.0 vs. 2.2 ± 1.5, *P* <0.001), male gender (87.7% vs. 92.6%, *P* = 0.016), age (48.4 ± 7.8 years vs. 45.7 ± 8.1 years, *P* <0.001), polypharmacy (12.5% vs. 7.4%, *P* <0.001), dipeptidyl peptidase-4 inhibitor use (37.5% vs. 30.6%, P = 0.031), and biguanide use (12.5% vs. 16.9%, *P* = 0.033).

**Table 1:** Patient characteristics at baseline.

	Overall n = 884	Nonadherent^a^ n = 444	Adherent^a^ n = 440	P (nonadherent vs. adherent)
Number of drugs^b^	2.4 ± 1.8	2.2 ± 1.5	2.6 ± 2.0	<0.001
Male, n (%)	797 (90.2)	411 (92.6)	386 (87.7)	0.016
Age (years)^b^	47.0±8.1	45.7 ± 8.1	48.4 ± 7.8	<0.001
Polypharmacy, n (%)^c^	88 (10.0)	33 (7.4)	55 (12.5)	<0.001
HbA1c (%)^b^	7.9 ± 2.0	7.9 ± 2.0	8.0 ± 1.9	0.384
BMI (kg/m^2^)^b^	26.5 ± 4.7	26.3 ± 4.4	26.6 ± 5.0	0.302
Hypoglycemic agent use rate, n (%)				
DPP-4 inhibitor	301 (34.0)	136 (30.6)	165 (37.5)	0.031
α-GI	208 (23.5)	109 (24.5)	99 (22.5)	0.473
Glinide	38 (4.3)	21 (4.7)	17 (3.9)	0.526
SU	204 (23.1)	101 (22.7)	103 (23.4)	0.815
Biguanide	235 (26.6)	104 (23.4)	131 (29.8)	0.033
Thiazolidine	130 (14.7)	75 (16.9)	55 (12.5)	0.065
SGLT-2 inhibitor	0 (0)	0 (0)	0 (0)	

^a^Patients were considered adherent if the proportion of days covered was ≥0.8 was considered adherent and nonadherent if this number was <0.8

^b^Values are presented as mean ± standard deviation.

^c^Polypharmacy is defined as taking ≥5 drugs.

Abbreviations: HbA1c: hemoglobin A1c, BMI: body mass index, DPP-4 inhibitor: dipeptidyl peptidase-4 inhibitor, α-GI: α-glucosidase inhibitor, SU: sulfonylurea, SGLT2 inhibitor: sodium glucose cotransporter 2 inhibitor.

Table 2 depicts the details of the mean PDC and adherence, depending on the patients’ background characteristics, at the 3-year observation period endpoint. The 49.8% of subjects were classified as adherent, and about half nonadherent. The total number of visits by all subjects for the purpose of diabetes treatment during the 3-year observation period was 24.1 ± 16.0 times, and ROC analysis revealed that this number had a cut-off of 17 times (sensitivity 95.9%, specificity 60.6%) and an area under the curve of 0.86.

**Table 2:** Adherence in relation to percentage of PDC among patients with at least 3 year of follow-up.

	Adherent^a^ (%)	PDC (%) [Median (25%–75%)]
All patients	49.8	79.6 (31.7–96.1)
Sex		
Men	48.4	78.1 (31.7–95.8)
Woman	62.1	86.7 (34.0–98.9)
Age (years)		
<40	38.9	66.4 (24.5–93.0)
40 to <50	45.5	76.3 (28.8–95.2)
50 to <60	59.1	87.0 (42.5–97.6)
60≤	57.7	85.7 (38.2–95.9)
Number of medications		
<5	48.4	78.5 (29.6–95.7)
5≤	68.5	91.8 (53.0–98.8)
HbA1c (%)		
<7%	49.4	78.8(25.7–95.0)
7%≤	54.4	84.8(45.1–96.9)
BMI (kg/m^2^)		
<25	55.2	84.9 (38.3–97.6)
25≤	49.1	79.0 (31.9–95.2)
DPP-4 inhibitor		
No	47.2	76.5 (29.2–95.3)
Yes	54.8	85.5 (38.2–97.8)
α-GI		
No	50.4	80.4 (35.6–96.2)
Yes	47.6	77.2 (25.1–95.8)
Glinide		
No	51.1	79.9 (32.4–96.1)
Yes	44.7	68.4 (18.1–96.6)
SU		
No	49.6	79.4(29.3–96.0)
Yes	50.5	80.1(46.7–96.4)
Biguanide		
No	47.6	76.9 (29.3–95.6)
Yes	55.7	85.1 (40.0–97.3)
Thiadolizine		
No	51.1	81.0 (31.7–96.7)
Yes	42.3	72.3 (32.2–93.8)

^a^Patients were considered adherent if the proportion of days covered was ≥0.8.

Abbreviations: HbA1c: hemoglobin A1c, BMI: body mass index, DPP-4 inhibitor: dipeptidyl peptidase 4 inhibitor, α-GI: α- glucosidase inhibitor, SU: sulfonylurea, PDC: proportion of days covered

The results of logistic regression analysis using adherence as the response variable are shown in Table 3. These results for the following variables were statistically significant: number of medications 3–4 (OR 1.68, 95% confidence interval (CI) 1.07–2.64, *P* = 0.024) or ≥5 (OR 2.74, 95% CI 1.38–5.46, *P* = 0.004), male sex (OR 0.45, 95% CI 0.23–0.89, *P* = 0.022), age ≥50 and <60 years (OR 2.15, 95% CI 1.15–3.99, *P* = 0.016), and total number of visits ≤17 (OR 29.9, 95% CI 18.4–48.7, *P* < 0.001).

**Table 3:** Logistic regression analysis for factors related to adherence.

	Univariate analysis			Multivariate analysis		
	Odds ratio	95% CI	p	Odds ratio	95% CI	p
Number of medications						
1–2	Reference	-	-	Reference	-	-
3–4	1.62	1.19–2.23	0.003	1.68	1.07–2.64	0.024
≥5	2.03	1.28–3.22	0.003	2.74	1.38–5.46	0.004
Sex						
Female	Reference	-	-	Reference	-	-
Male	0.57	0.36–0.90	0.017	0.45	0.23–0.89	0.022
Age (years)						
<40	Reference	-	-	Reference	-	-
40 to <50	1.31	0.89–1.93	0.174	1.23	0.69–2.22	0.481
50 to <60	2.27	1.51–3.41	<0.001	2.15	1.15–3.99	0.016
60≤	2.14	1.12–4.08	0.020	1.67	0.66–4.21	0.278
Number of visits/3 years						
<17	Reference	-	-	Reference	-	-
17≤	24.0	15.7–36.6	<0.001	29.9	18.4–48.7	<0.001
HbA1c (%)						
7.0≤	Reference	-	-	-	-	-
<7.0	0.82	0.60–1.12	0.214	-	-	-
BMI (kg/m^2^)						
<25	Reference	-	-	Reference	-	-
25≤	0.79	0.59–1.05	0.108	0.86	0.58–1.27	0.446
DPP-4 inhibitor						
No	Reference	-	-	Reference	-	-
Yes	1.36	1.03–1.80	0.031	1.21	0.80–1.84	0.370
α-GI						
No	Reference	-	-	-	-	-
Yes	0.89	0.65–1.22	0.473	-	-	-
Glinide						
No	Reference	-	-	-	-	-
Yes	0.81	0.42–1.56	0.526	-	-	-
SU						
No	Reference	-	-	-	-	-
Yes	1.04	0.76–1.42	0.23	-	-	-
Biguanide						
No	Reference	-	-	Reference	-	-
Yes	1.39	1.03–1.87	0.033	1.48	0.93–2.36	0.096
Thiazolidine						
No	Reference	-	-	Reference	-	-
Yes	0.70	0.48–1.02	0.066	0.62	0.35–1.13	0.123

Abbreviations: HbA1c: hemoglobin A1c, BMI: body mass index, DPP-4 inhibitor: dipeptidyl peptidase-4 inhibitor, α-GI: α-glucosidase inhibitor, SU: sulfonylurea

The average HbA1c value at the observation endpoint was 7.2 ± 1.4% (mean ± SD), and 52.3% of the subjects achieved an HbA1c value of <7.0%. The results of multivariate analysis (Table 4) indicated that the following factors were statistically significant in achieving HbA1c levels <7.0% at the end of the observation period: adherence (OR 1.84, 95% CI 1.28–2.66, *P* = 0.001), age ≥50 and <60 years (OR 2.34, 95% CI 1.30–4.21, *P* = 0.005), HbA1c value of <7.0% (OR 5.01, 95% CI 3.36–7.47, *P* < 0.001), and sulfonylurea use (OR 0.54, 95% CI 0.34–0.86, *P* = 0.009).

**Table 4:** Factors related to the achievement of HbA1c value of <7.0% at the end of the 3-year of follow-up observation period.

	Univariate analysis			Multivariate analysis		
	Odds ratio	95% CI	P	Odds ratio	95% CI	P
Number of medications						
1–2	Reference	-	-	Reference	-	-
3–4	1.25	0.89–1.75	0.203	1.03	0.66–1.60	0.911
5≤	1.88	1.15–3.07	0.012	1.03	0.56–1.89	0.925
Medication						
Nonadherence	Reference	-	-	Reference	-	-
Adherence	1.49	1.12–1.97	0.006	1.84	1.28–2.66	0.001
Sex						
Female	Reference	-	-	-	-	-
Male	1.12	0.70–1.79	0.633	-	-	
Age (years)						
<40	Reference	-	-	Reference	-	-
40 to <50	1.15	0.77–1.73	0.498	1.33	0.76–2.32	0.325
50 to <60	2.13	1.39–3.28	0.001	2.34	1.30–4.21	0.005
≥60	2.01	0.98–4.12	0.057	2.11	0.86–5.21	0.103
Number of visits/3 years						
<17	Reference	-	-	-	-	-
≥17	1.06	0.79–1.42	0.702	-	-	-
HbA1c (%)						
≥7.0	Reference	-	-	Reference	-	-
<7.0	5.34	3.68–7.73	<0.001	5.01	3.36–7.47	<0.001
BMI (kg/m^2^)						
<25	Reference	-	-	Reference	-	-
≥25	0.73	0.55–0.98	0.037	0.98	0.67–1.43	0.922
DPP-4 inhibitor						
No	Reference	-	-	Reference	-	-
Yes	1.00	0.74–1.35	0.01	0.85	0.57–1.28	0.439
α-GI						
No	Reference	-	-	Reference	-	-
Yes	1.59	1.14–2.23	0.007	1.28	0.50–2.06	0.300
Glinide						
No	Reference	-	-	Reference	-	-
Yes	0.38	1.78–0.80	0.011	0.38	0.14–1.03	0.057
SU						
No	Reference	-	-	Reference	-	-
Yes	0.40	0.28–0.57	<0.001	0.54	0.34–0.86	0.009
Biguanide						
No	Reference	-	-	-	-	-
Yes	0.81	0.59–1.12	0.202	-	-	-
Thiazolidine						
No	Reference	-	-	-	-	-
Yes	1.10	0.74–1.62	0.635	-	-	-

Abbreviations: HbA1c: hemoglobin A1c, BMI: body mass index, DPP-4 inhibitor: dipeptidyl peptidase-4 inhibitor, α-GI: α-glucosidase inhibitor, SU: sulfonylurea, PDC: proportion of days covered

## Discussion

Our results indicate that PDC for the 3-year observation endpoint was 79.6%. In only 49.8% of the cases we observed a PDC ≥0.8; thus, about half of the patients were nonadherent. The relatively higher medication adherence in our study than that in previous studies [3–5], may be attributable to the fact that we had an observation period of three years. A short observation period of only one year in previous studies might have resulted in less chances to improve medication adherence due to the loss of some opportunities where interventions could have been made if a longer period of observation was considered.

Hence, we believe that it is necessary to make efforts to improve medication adherence in order to achieve the target blood glucose level and to control the onset of diabetes complications.

Our study has revealed several factors related to adherence. We observed a positive relationship between adherence and number of visits ≥17 during the 3-year period. Frequent visits provide opportunities for physicians and pharmacists to re-evaluate prescriptions according to the patient’s current condition. The present study demonstrated that polypharmacy and older age were associated with adherence, which is consistent with the results of a previous study [18]. Several studies that assessed patients with T2DM with a high number of medications [19] and complications from other chronic diseases [20–23] have shown a positive association between polypharmacy and adherence. The condition of polypharmacy reportedly leads to various complications, and is believed that medication adherence is higher in patients with polypharmacy compared to those with a small number of medications. In contrast, if there are any subjective symptoms due to complications, medication adherence may be increased to improve them.

The relationship between gender and medication adherence is controversial [24–27]. In Japan, treatment interruptions in men under 40 years of age with T2DM are higher and medication adherence is reportedly low [28]. We also observed a similar trend, which is probably due to the fact that most of the cases included in our study fell in a similar age range. We also observed that medication adherence improved with age until the age of 60 years, as we did not observe any significant difference in the OR values in patients older than 60 years. In a previous adherence study in Japan [29], the mean PDC and the percent of adherent patients with T2DM had reportedly improved with the increase in the number of concomitant medications, with the highest PDC observed among patients on three or more concomitant medications. The lowest PDC was observed in patients who were only taking medication for T2DM [29]. Although it has been shown that the odds ratio for polypharmacy increases with the older age [30], we did not observe any significant improvement in adherence in the ≥60 age group. This result might be due to the smaller number of cases in the ≥60 age group compared to other age groups in our cohort. Finally, we investigated the factors related to achievement of an HbA1c value of <7.0% at the end of the 3-year observation period. The present study indicated that adherence has a positive effect on the achievement of an HbA1c value of <7.0%, and further investigation of its effect on the risk of diabetic complications is warranted. With adherence, hypoglycemic administration can be expected to be more effective, which in turn is likely to lead to satisfactory HbA1c levels being recorded at the observation endpoint. Although we found that being in the age group ≥50 to <60 years had a positive effect on achievement of the HbA1c level <7.0%, despite the general increase in insulin resistance and decrease in insulin production as a person ages, which may lead to a rise in HbA1c levels. We attribute the positive effect we observed in our study to the fact that medication adherence in this age group was generally good. However, we did not further elucidate the details of this particular aspect. Prescribing sulfonylureas has been reportedly related with older age, long-standing disease, and poor glycemic control [31]. The results of the present study indicated that sulfonylurea use was a risk factor for non-achievement of the HbA1c target of <7.0% because it tends to be used positively in cases that are having difficulty in glycemic control.

Our study has several limitations due to its retrospective nature and the use of itemized medical claims, which will lead to some difficulties in eliminating data and selection biases. We used the information available on Japan Medical Data Center database, which includes claims from employer-sponsored healthcare plans. Hence, our cohort only consisted of adults who were employed. The database includes information on a small number of diabetic patients≥75 years, which were excluded from this study. Nonetheless, previous studies have reported that elderly patients have better medication adherence than younger patients [18,28,32]. Therefore, while we might expect that medication adherence and persistence would be higher in older Japanese patients, further research is required to confirm this hypothesis. We did not collect other data that may have affected medication adherence, such as financial difficulties [33], ethnicity [34,35], psychological factors [36], and the quality of the relationship between patient and physician [37]. Finally, most of the subjects in this study were male; therefore, in order to generalize the findings it is necessary to conduct further studies which include a higher percentage of female patients with T2DM. We plan to conduct a detailed study with a large cohort in the near future.

## Conclusions

In this study, we found that older age groups, taking several medications, and regular visits are associated with a better adherence. We also elucidated the effect of adherence on achievement of satisfactory HbA1c levels at the observation endpoint. Because maintaining medication adherence and achieving HbA1c targets are important in reducing the likelihood of diabetic complications, targeted interventions for different groups–such as those of younger age and taking fewer medications–need to be developed.
